# Supplementary material for: The Flipped Break-Even: Re-Balancing Demand- and Supply-Side Financing of Health Centers in Cambodia
Source: Int J Environ Res Public Health. 2023 Jan 10;20(2):1228. doi: 10.3390/ijerph20021228 (PMC9858853; doi:10.3390/ijerph20021228)
Supplement: Supplementary file 1 [file ijerph-20-01228-s001.zip › ijerph-2056944-supplementary.pdf]

Table S1. Basic descriptive statistics.

HC Code	Cost per Service Unit [US$]	Quality Score 2019	Margin [US$]	Efficiency
1	4.01	81.51%	0.91	0.1771506
2	5.48	69.71%	1.73	0.1382938
3	6.49	84.22%	3.04	0.1635298
4	3.73	77.19%	1.09	0.2512313
5	8.33	84.06%	2.15	0.1243696
6	5.79	81.50%	2.07	0.1735644
7	5.08	80.64%	1.08	0.2236639
8	2.96	82.52%	0.87	0.3199978
9	4.57	81.22%	1.69	0.2344077
10	5.42	78.69%	1.66	0.1692028
11	10.09	78.60%	1.84	0.1199557
12	6.77	69.48%	1.49	0.1282886
13	5.29	74.56%	1.50	0.1843995
14	6.09	69.04%	1.69	0.1752705
B15	9.70	76.90%	2.20	0.093766
B16	3.97	85.82%	1.20	0.248127
B17	5.30	78.46%	0.78	0.1724847
18	7.28	63.39%	1.52	0.0885271
19	4.09	63.92%	0.99	0.1464789
20	6.29	80.86%	1.24	0.1357024
21	4.14	56.25%	1.09	0.1383619
22	8.38	59.32%	2.18	0.0656015
23	3.25	69.27%	0.61	0.2275223
24	4.95	70.86%	1.03	0.2164801
25	6.29	69.40%	1.25	0.1164698
26	5.51	49.15%	1.06	0.0974757
27	6.34	74.57%	1.30	0.1426906
28	5.31	63.33%	1.53	0.1403057
29	2.94	72.12%	0.54	0.2949239
30	3.49	76.38%	1.02	0.2475647
31	6.26	66.16%	1.28	0.1103088
32	3.38	65.66%	0.73	0.2611843
33	19.66	48.57%	1.70	0.0263169
34	3.94	64.82%	1.02	0.1673303
35	11.31	71.03%	2.59	0.1045601
36	3.50	62.06%	0.81	0.2396742
K1	10.95	84.02%	2.46	0.1057895
K2	12.82	78.30%	3.55	0.0967823
K3	8.06	87.42%	2.92	0.1332217
K4	10.75	91.76%	5.20	0.0789176
K5	12.32	79.20%	6.45	0.0745052
K6	8.77	84.90%	2.83	0.1448091
K7	15.22	88.88%	6.52	0.0644579
K8	11.68	81.13%	2.88	0.1139469
K9	9.57	83.83%	2.69	0.1403089
K10	15.01	71.04%	5.52	0.0406728
K11	8.49	73.83%	3.46	0.0998125
K12	8.78	70.71%	2.44	0.1508249
K13	7.00	80.52%	2.08	0.1515459

HC Code	Cost per Service Unit [US$]	Quality Score 2019	Margin [US$]	Efficiency
K14	8.67	79.22%	2.47	0.1481134
K15	9.91	74.82%	3.46	0.104469
K16	10.55	73.99%	4.97	0.0889251
K17	9.58	80.31%	3.82	0.1062087
K18	12.85	77.02%	5.18	0.0919072
K19	18.98	71.10%	9.55	0.0404901
K20	17.43	70.34%	8.33	0.0370014
K21	10.48	71.69%	3.58	0.0641377

Source: [39], own.

Table S2. Health Center Balanced Score Card (Structural Quality).

	Indicator	Criterion	Weight
1.1	**Financial Management**		**Max: 10 points**
1.1.1	Health Center Management Committee	1. Annual HCMC meetings schedule is available<br>2. Minutes of meetings are present. Minutes follow template<br>3. Financial report was discussed<br>4. Having quarterly achievement report of the previous quarter compared to the set target of at least 4 departments (OPD, ANC, delivery, immunization)	2
1.1.2	Expense Register	1. Income and Expense register is available<br>2. daily income and expenses are calculated<br>3. Three randomly selected daily income and expense calculations are correct	1
1.1.3	Monthly Income and Expense Statement	1. Monthly income and expense statement for the past month is available<br>2. Totals add up<br>3. There is proof that the monthly income expense statement is presented and discussed during the health center monthly meeting	2
1.1.4	Individual Performance Management	1. Individual performance management is done at the least once per quarter for each staff (All staff will be evaluated except Chief of HC)<br>2. Results of latest performance evaluations are used to allocate individual performance bonuses	3
1.1.5	Financial Management Tool	1. Quarterly financial report with total income, expenses and balance available for the past quarter.<br>2. Income and expense projection for the coming quarter available quoted from AOP.<br>3. Performance Grant: Performance budget of the last quarter (maximum 80% of the performance grant) was distributed based on each individual performance evaluation (check the performance budget distribution of the last quarter)<br>4. Random staff member interviewed knows (a) her/his bonus of the last quarter; (b) her/his last individual performance evaluation score and (c) can explain how her/his individual performance was evaluated.	3

	Indicator	Criterion	Weight
1.2	**HEF Management**		**Max: 2 Points**
1.2.1	Counting the number of HEF cases over the past quarter	1. Number of HEF cases recorded in the register books with a HEF card number during one month over the past quarter	1
1.2.2	Inquire whether non-medical benefits have been provided	1. If Health Center provided transportation or food in the past quarter: check the total amount recorded on invoice for HEF reimbursement and total amount recorded on the supporting document.	1

	Indicator	Criterion	Weight
1.3	**Infection control, Hygiene and Medical Waste Disposal**		**Max: 15 Points**
1.3.1	General Premises	1. The health center has a surrounding wall or fence<br>2. The premises do not have medical waste (wrappers or needles lying around). (A single medical waste item anywhere on the premises leads to a zero score)<br>3. The premises have no litter of any kind lying around (paper; plastic; a single paper or plastic wrap anywhere on the premises lost only 0.25 point)<br>4. The premises have at least one clearly visible garbage can not full.	**1**
1.3.2	Cleanliness	Assess each Ward: OPD; Delivery room; and ANC room: 3 in total<br>1. The floor, ceiling, wall, window and door are clean and there is no litter on the floor.<br>2. The ward smells of disinfectant<br>3. The ward has at least one garbage bin, which is not full (more litter can still be added without spilling over).	**2**
1.3.3	Hand washing supplies	Assess OPD; delivery room; and ANC room<br>1. Running water present (tap is functioning, and water is coming out) or container with a functioning tap containing water<br>2. Paper napkins or clean clothes available to dry hands<br>3. Soap is available (detergent not allow)<br>4. Alcohol for hand washing available	**2**

	Indicator	Criterion	Weight
1.3.4	Toilet facilities	1. Presence of two toilets<br>2. The toilet has a door and each toilet is lockable from the inside<br>3. Each lock is functional<br>4. Each door cannot be locked from the outside<br>5. Each toilet has running water or container with water with a ladle for flushing the toilet<br>6. Each toilet has a place (outside next to toilet) to wash hands with running water - or a water container with functioning tap containing water- , paper napkins or clean clothes and soap<br>7. Each toilet has a light (either electric or solar)<br>8. Each toilet has no visible waste<br>9. Each toilet has no flies<br>10. Each toilet smells of disinfectant or air refresher	**2**
1.3.5	Shower facilities	1. Presence of at least one shower with running water or water container with water and ladle containing water (full or at the least about half full) or a big water container for shower and the small container for flushing the toilet.<br>2. The room is lockable from the inside, and the lock is functional and the room cannot be locked from the outside<br>3. The shower has an exhaust fan or ventilation through tiles	**1**
1.3.6	Medical waste Handling	1. Assess delivery room; ANC room; OPD and immunization room<br>2. Rubbish bin is available and not full<br>3. Rubbish bin does not contain used syringes or needles<br>4. Safety Box/Sharps container available and not full (sharps container need if the room use syringes or needles only).	**1**
1.3.7	Disposal of health care waste	1. Medical Waste Management Plan is available, designated staff is there; four elements described (sorting; handling; interim storage; final disposal)<br>2. Medical waste and general waste are sorted and disposed in containers and plastic bags with color and logo according to the 2017 HCWM guidelines.<br>3. Sharps waste and needles are disposed in safety boxes for burning in high temperature (more than 800 degree C) incinerator. The safety boxes which are full of sharp waste (not to exceed the safety box marking) must not exceed 3 boxes in the health center compound.<br>4. Record safety boxes which are sent to high temperature (more than 800° C) incinerator.<br>5. Infectious medical waste is either burnt or buried safely according to the 2017 HCWM guidelines.<br>6. Placenta pit available for placenta disposal.	**6**

Source: Ministry of Health, Cambodia. 2022.
